# Ghrelin and eating behavior: evidence and insights from genetically-modified mouse models

**DOI:** 10.3389/fnins.2013.00121

**Published:** 2013-07-16

**Authors:** Aki Uchida, Jeffrey M. Zigman, Mario Perelló

**Affiliations:** ^1^Divisions of Hypothalamic Research and Endocrinology and Metabolism, Department of Medicine, The University of Texas Southwestern Medical CenterDallas, TX, USA; ^2^Department of Psychiatry, The University of Texas Southwestern Medical CenterDallas, TX, USA; ^3^Laboratory of Neurophysiology of the Multidisciplinary Institute of Cell Biology [Argentine Research Council (CONICET) and Scientific Research Commission, Province of Buenos Aires (CIC-PBA)]La Plata, Buenos Aires, Argentina

**Keywords:** hedonic eating, homeostatic eating, GOAT, GHSR

## Abstract

Ghrelin is an octanoylated peptide hormone, produced by endocrine cells of the stomach, which acts in the brain to increase food intake and body weight. Our understanding of the mechanisms underlying ghrelin's effects on eating behaviors has been greatly improved by the generation and study of several genetically manipulated mouse models. These models include mice overexpressing ghrelin and also mice with genetic deletion of ghrelin, the ghrelin receptor [the growth hormone secretagogue receptor (GHSR)] or the enzyme that post-translationally modifies ghrelin [ghrelin O-acyltransferase (GOAT)]. In addition, a GHSR-null mouse model in which GHSR transcription is globally blocked but can be cell-specifically reactivated in a Cre recombinase-mediated fashion has been generated. Here, we summarize findings obtained with these genetically manipulated mice, with the aim to highlight the significance of the ghrelin system in the regulation of both homeostatic and hedonic eating, including that occurring in the setting of chronic psychosocial stress.

## Introduction

Ghrelin is a 28-amino acid octanoylated peptide hormone secreted predominantly from a distinct population of endocrine “ghrelin” cells located within the gastric oxyntic mucosa, previously referred to as P/D1-type cells in humans and A like-type cells in rodents (Kojima and Kangawa, 2010). Ghrelin is the only known naturally occurring peptide to be modified by Ser^3^ O-octanoylation, a reaction catalyzed by the enzyme ghrelin O-acyl transferase (GOAT) (Yang et al., 2008; Kojima and Kangawa, 2010). This post-translational modification is essential for the binding of ghrelin to and subsequently activation of its specific receptor, the growth hormone secretagogue receptor type 1a (GHSR). GHSR is a 7 transmembrane protein coupled to the Gq subfamily of heterotrimeric GTP-binding proteins that activates phospholipase C and in turn, induces release of calcium from intracellular stores (Howard et al., 1996; Damian et al., 2012). Stimulation of other signal transduction cascades also have been demonstrated upon activation of GHSR, although much of that data was characterized using transfected cell systems (Cruz and Smith, 2008). GHSR is expressed in several regions of the central nervous system and some peripheral organs (Cruz and Smith, 2008).

Ghrelin's actions are diverse. Ghrelin is a potent growth hormone (GH) secretagogue and orexigenic peptide (Kojima and Kangawa, 2010). Ghrelin also prevents falls in blood glucose upon extreme caloric restriction and minimizes depressive-like behavior upon chronic psychosocial stress (Lutter et al., 2008; Goldstein et al., 2011). Moreover, ghrelin stimulates motor activity in the gastrointestinal tract and accelerates gastric emptying (Peeters, 2003). Although desacyl-ghrelin, the non-octanoylated form, does not bind to GHSR, some studies report that it can modify certain physiological responses, including food intake and glucose homeostasis (Delhanty et al., 2012).

Genetically-modified mouse models have been instrumental in dissecting out the physiological roles of ghrelin, as well as the specific sites of ghrelin action. Genetic modifications in mice can be grouped in two classes: (1) Standard transgenic models, in which a foreign DNA construct is randomly integrated into the mouse genome (commonly used for overexpression models) and (2) Gene-targeted transgenic models, in which a gene disruption or manipulation is introduced in a single gene via homologous recombination between the foreign DNA and the endogenous gene (commonly used for “knockout” and “knock-in” models) (Davey and Maclean, 2006). In this review, we discuss ghrelin's action on eating behaviors as determined using mice with genetic manipulations in the ghrelin signaling system (Tables 1 and 2). We first focus on the role of ghrelin in homeostatic eating and subsequently discuss the role of ghrelin in hedonic eating.

**Table 1:** **Studies of homeostatic eating behaviors in mouse models with genetic alterations in ghrelin-signaling system**.

**Model**	**Summary**	**References**
Ghrelin overexpression (driven by various promoters)	Elevated desacyl-ghrelin levels with conflicting food intake and body weight results	Ariyasu et al., 2005; Asakawa et al., 2005; Iwakura et al., 2005; Wei et al., 2006; Zhang et al., 2008
	Elevated ghrelin levels with no change in food intake (Reed et al.) or increase in food intake (Bewick et al.)	Reed et al., 2008; Bewick et al., 2009
Ghrelin overexpression (SV40 T-antigen)	Increased ghrelin levels, with food intake and body weights indistinguishable from wild-type littermates; Older transgenic mice lose weight, likely due to large gastric tumors	Iwakura et al., 2009; Zhao et al., 2010b
Ghrelin analog overexpression	Increase of ghrelin-like activity, no change in food intake, body weight, energy expenditure or fat mass	Matsumoto et al., 2001; Yamada et al., 2010; Ariyasu et al., 2012
Human ghrelin and GOAT overexpression	Elevated human ghrelin only when fed diet containing medium-chain triglycerides. No change in food intake but increase in body weight	Kirchner et al., 2009
Ghrelin knockout	No changes in eating behaviors under CHD. Inconsistent results in HFD feeding studies	Sun et al., 2003; Wortley et al., 2004, 2005; De Smet et al., 2006; Dezaki et al., 2006; Pfluger et al., 2008; Sato et al., 2008
GHSR knockout	Attenuated anticipatory locomotor response. Similar food intake, but subtle decrease in body weight under CHD feeding	Sun et al., 2004, 2008; Abizaid et al., 2006; Blum et al., 2009; Lin et al., 2011; Ma et al., 2011
GHSR null (removable transcriptional blocking cassette)	Female mice on CHD weigh less than wild-type controls. Consume less food and are resistant to HFD-induced body weight gain upon early HFD exposure	Zigman et al., 2005; Perello et al., 2012
Ghrelin and GHSR knockout	Lower body weight, but no change in food intake	Pfluger et al., 2008
Site-selective GHSR Expression (GHSR null crossed with cell-specific Cre)	TH-promoter driven Cre: partially restores ghrelin-stimulated food intake Phox2b-promoter driven Cre: lack ghrelin-induced food intake	Chuang et al., 2011; Scott et al., 2012
GHSR overexpression in GHRH neurons	Increase in post-weaning growth rate. Increase in growth rate not maintained in adulthood. No change in body weight on HFD, although fat pad mass trended lower. Possible increase in HFD intake	Lall et al., 2004
GOAT deficiency	Normal body weight and fat mass under CHD feeding. Increase in food intake but lower body weights under medium-chain triglyceride rich diet	Gutierrez et al., 2008; Kirchner et al., 2009; Zhao et al., 2010a
Ghrelin knockout (*Ob/Ob* background)	Similar food intake and a modest increase in body weight as compared to ob/ob mice	Sun et al., 2006
GHSR knockout (*Ob/Ob* background)	Similar food intake and body weights to ob/ob mice	Ma et al., 2012

**Table 2:** **Studies of hedonic eating behaviors in mouse models with genetic alterations in ghrelin-signaling system**.

**Model**		**References**
GHSR knockout	Suppressed intake of rewarding food in a free choice (CHD/rewarding food) paradigm	Blum et al., 2009; Egecioglu et al., 2010; Disse et al., 2011; Verhagen et al., 2011; Lamont et al., 2012
GHSR null	Lack cue potentiated feeding behavior. Do demonstrate CPP for HFD post-CSDS. Fail to increase intake of CHD in response to chronic stress	Perello et al., 2010; Chuang et al., 2011; Walker et al., 2012
Site-selective GHSR expression (GHSR null crossed with cell-specific Cre)	TH-promoter driven Cre: sufficient to restore CSDS-induced CPP for HFD	Chuang et al., 2011
GOAT deficiency	Attenuated motivation for food in an operant responding model. Decreased hedonic feeding response as examined in a “dessert effect” protocol	Davis et al., 2012

## Ghrelin and homeostatic eating

Homeostatic eating is defined as food intake driven by necessity, due to energy deficiency as perceived by the brain and body. Ghrelin is the only known circulating peptide hormone that stimulates food intake (Cummings, 2006). Ghrelin triggers eating even at times of minimal spontaneous food intake, and its orexigenic actions are rapid, short-lived and potent (Nakazato et al., 2001; Wren et al., 2001; Cummings, 2006). It has been suggested that ghrelin is a physiologic meal initiator, since its levels increase preprandially and decrease post-prandially (Cummings et al., 2001). Ghrelin levels, in individuals under restricted feeding schedules, increase approximately 1 h prior to food presentation. In animals, this preprandial rise in ghrelin occurs simultaneously or possibly precedes the increased locomotion in anticipation of a scheduled meal (Drazen et al., 2006). Indeed, ghrelin administration increases locomotor activity in rats and foraging-like activities in hamsters (Keen-Rhinehart and Bartness, 2005; Jerlhag et al., 2007). Thus, Blum et al. hypothesized that ghrelin is secreted in anticipation of a meal and correlates with anticipatory locomotor activity (Blum et al., 2009).

The mechanism for ghrelin's action on stimulating food intake was initially thought to be through homeostatic hypothalamic circuits originating in the arcuate nucleus (ARC) (Willesen et al., 1999; Nakazato et al., 2001; Kageyama et al., 2010). The expression of GHSR, the only known receptor for ghrelin, has been confirmed within the ARC by many techniques, including *in situ* hybridization histochemistry, RT-PCR, immunohistochemistry and Western blot analysis (Howard et al., 1996; Guan et al., 1997; Willesen et al., 1999; Zigman et al., 2006). Several studies have demonstrated that ghrelin has the capacity to bind its receptor on arcuate NPY/AgRP neurons, resulting in their activation and ensuing orexigenic behaviors (Willesen et al., 1999; Nakazato et al., 2001; Wren et al., 2001; Kageyama et al., 2010; Schaeffer et al., 2013). Interaction of ghrelin with several other neuronal subtypes and brain sites also has been shown to increase intake of freely-available food. These include, among others, orexin-producing neurons of the lateral hypothalamus (Olszewski et al., 2003; Toshinai et al., 2003). Additionally, some evidence indicates that vagus nerve integrity is required for ghrelin-induced food intake as well (Date et al., 2002; Date, 2012).

### Homeostatic eating in mice over expressing ghrelin

In order to confirm the effects of ghrelin on homeostatic eating, several groups have attempted to produce transgenic mice with chronically elevated ghrelin. These initial transgenes contained the ghrelin gene driven by various promoters—including those for CAG (Ariyasu et al., 2005; Asakawa et al., 2005), rat insulin II (Iwakura et al., 2005), rat glucagon (Iwakura et al., 2005), CMV (Wei et al., 2006) and FABP4 (Zhang et al., 2008)—resulting in elevated ghrelin gene expression mainly in cell types in which ghrelin gene expression does not usually occur. These mouse models resulted in elevated desacyl-ghrelin rather than elevations of ghrelin, presumably because this overexpression of the ghrelin gene was targeted mostly to tissues lacking the ghrelin-acylating enzyme, GOAT. Indeed, GOAT was not discovered until 2008. Most mouse models overproducing desacyl-ghrelin exhibited similar food intake and body weight as compared to wild-type mice (Iwakura et al., 2005; Wei et al., 2006); nonetheless, some studies found reduced body weight, fat mass, nose-to-anus length and/or food intake in their transgenic mice overexpressing desacyl-ghrelin (Ariyasu et al., 2005; Asakawa et al., 2005; Zhang et al., 2008). These findings may suggest that desacyl-ghrelin has metabolic effects opposite to those of ghrelin.

Two groups successfully produced standard transgenic mice with ghrelin overexpression. Reed et al. generated a transgenic mouse model overexpressing ghrelin in neurons by using a neuron-specific enolase promoter. These mice showed increased ghrelin expression in brain and increased circulating ghrelin (~5-fold), although they did not differ from wild-type controls in body weight, food intake, energy expenditure or fat mass (Reed et al., 2008). Likewise, Bewick et al. generated a transgenic mouse model overexpressing ghrelin, using a bacterial artificial chromosome containing the ghrelin gene and its promoter (Bewick et al., 2009). These mice exhibited increased circulating ghrelin (~1.5-fold) and increased food intake without long-term body weight gain, perhaps due to a paradoxical increase in energy expenditure (Bewick et al., 2009). Bewick et al. proposed that the differences between their model and the Reed et al. mouse model could be due to differential peripheral vs. central nervous system ghrelin concentrations (Bewick et al., 2009). Of note, results regarding the existence, sites of production and physiological significance of endogenous central ghrelin production in non-genetically-manipulated models have been inconsistent (Furness et al., 2011), although ghrelin from the periphery does make its way to the central nervous system.

After GOAT was identified, Kirchner et al. generated a double-transgenic mouse model overexpressing both human ghrelin and MBOAT4 (the membrane-bound O-acyltransferase domain containing 4 or human variant of GOAT) genes in the liver via the human apo-lipoprotein E promoter (Kirchner et al., 2009). These transgenic mice, which exhibited elevated concentrations of human ghrelin only when fed on a diet containing medium-chain triglycerides, showed decreased energy expenditure and increased rate of body weight gain without changes in food intake (Kirchner et al., 2009).

Other unique techniques have been employed to increase ghrelin expression. Yamada and colleagues generated transgenic mice overexpressing a ghrelin analog, which replaced Ser^3^ with Trp^3^, under the control of human serum amyloid-P promoter (Yamada et al., 2010). Trp^3^-ghrelin possesses lower levels of ghrelin-like activity without the need for post-transcriptional modification (Matsumoto et al., 2001). Although mice overexpressing Trp^3^-ghrelin displayed a ~6-fold increase of ghrelin-like activity, neither body weight, food intake, energy expenditure or fat mass differed from wild-type controls (Ariyasu et al., 2012). Our group generated transgenic mice that express the immortalizing SV40 large T-antigen in ghrelin cells (Zhao et al., 2010b). These mice develop ghrelin-secreting tumors, which result in ~25-fold higher plasma ghrelin concentrations and ~37-fold higher plasma desacyl-ghrelin levels by the time they reach 20 weeks of age (Zhao et al., 2010b). Despite the sustained increase in plasma ghrelin levels, food intake and body weights in these transgenic mice were indistinguishable from those in wild-type mice; older transgenic mice lost weight, presumably due to the development of large gastric tumors (Zhao et al., 2010b). Iwakura et al. also created ghrelin promoter SV40 large T-antigen transgenic mice (Iwakura et al., 2009). These mice showed no difference in body weights as compared to wild-type mice before 12 weeks of age, after which body weight and food intake declined, likely due to tumor growth (Iwakura et al., 2009).

### Homeostatic eating in mice with ghrelin deficiency

To our knowledge, four different ghrelin knockout mouse models have been reported (Sun et al., 2003; Wortley et al., 2004; De Smet et al., 2006; Dezaki et al., 2006). Regardless of the complete absence of ghrelin in circulation in these models, under *ad libitum* standard rodent chow diet (CHD) feeding, food intake and body weights in these ghrelin knockout mice were indistinguishable from those in wild-type mice. In addition, no differences were observed when other parameters of food intake were measured, including post-fasting hyperphagia (Sun et al., 2003; Wortley et al., 2004; Pfluger et al., 2008), forced dark cycle-induced eating (De Smet et al., 2006) or eating memory (Sato et al., 2008). These findings suggest that genetic ghrelin deficiencies fail to cause significant alterations in homeostatic aspects of eating behaviors.

Furthermore, three of the four ghrelin knockout models have been studied under chronic high fat diet (HFD) feeding, but yielded inconsistent results (Wortley et al., 2005; Dezaki et al., 2006; Sun et al., 2008). Surprisingly, none of the studies exhibited the expected reduction of HFD intake. Wortley et al. did demonstrate that ghrelin deficiency resulted in reduced body weight and fat mass, but these results were not recapitulated in the other mouse ghrelin knockout mouse models. Differences in the age at which the mice were first exposed to HFD diet are thought to account for the inconsistencies in the body weight phenotype among the different ghrelin knockout models. In particular, resistance to the development of diet-induced obesity may manifest in ghrelin knockout mice upon early exposure to HFD, but not when HFD challenge is initiated later in life (Wortley et al., 2004, 2005; Sun et al., 2008).

### Homeostatic eating in mice with GHSR deficiency

To our knowledge, three different mouse models with GHSR deficiency have been reported in the literature (Sun et al., 2004; Zigman et al., 2005; Abizaid et al., 2006). Two are traditional GHSR knockout models, in which the GHSR gene has been removed (Sun et al., 2004; Abizaid et al., 2006), and the other is a GHSR-null model in which the GHSR locus has been modified by the insertion of a loxP-flanked transcriptional blocking cassette (Zigman et al., 2005). More specifically, this transcriptional blocking cassette sequence disrupts GHSR gene expression and, as a consequence, the GHSR-null mice (harboring 2 copies of the recombinant GHSR allele) have no detectable functional GHSR expression. Cre recombinase mediates reactivation of GHSR gene expression by removal of the transcriptional blocking cassette sequence.

GHSR-deficient mice show a subtle but significant decrease in body weight. Sun et al. reported that GHSR knockout mice fed on CHD show a lower body weight than wild-type mice from 16 to 24 weeks of age (Sun et al., 2004). GHSR-deficient mice do not differ from wild-type controls in food intake under CHD (Sun et al., 2004; Zigman et al., 2005). Sun et al. found that GHSR knockout mice have a similar susceptibility to diet-induced obesity compared to wild-type mice upon exposure to HFD as adults (Sun et al., 2008); however, GHSR knockout mice manifest reduced age-associated obesity mainly due to reduced adiposity and increased thermogenesis (Lin et al., 2011; Ma et al., 2011). Using a restrictive eating paradigm, Abizaid et al. showed that GHSR knockout mice did not increase their food intake in response to repeated fasts as seen in wild-type mice. This was also true in ghrelin knockout mice (Abizaid et al., 2006).

In the GHSR-null mice from our group, female GHSR-null mice on CHD were significantly lighter than wild-type controls starting at 12 weeks of age (Zigman et al., 2005). Also, in two independent studies, GHSR-null mice were resistant to HFD-induced body weight gain upon early HFD exposure (Zigman et al., 2005; Perello et al., 2012).

As previously mentioned, in animals, a preprandial rise in ghrelin occurs simultaneously or possibly precedes the increased locomotion exhibited in anticipation of a scheduled meal (Drazen et al., 2006). In preliminary studies, Gooley et al. examined food-entrainable circadian rhythms in a very small cohort of female GHSR-null mice (Gooley et al., 2006). Similar to wild-type littermates, the GHSR-null mice continued to show a preprandial rise in locomotor activity and body temperature, suggesting that intact ghrelin signaling was not required for the expression of food-entrainable circadian rhythms that are likely related to finding food (Gooley et al., 2006). Furthermore, Blum et al. also demonstrated increased activity prior to scheduled mealtime in both wild-type and GHSR knockout mice, although in their study, the response in GHSR knockout mice was attenuated (Blum et al., 2009). Others have also similarly reported that GHSR knockout mice under a restricted feeding paradigm demonstrate attenuated anticipatory locomotor responses and reduced expression of the marker of cellular activation c-fos in the mesolimbic dopamine pathway (Blum et al., 2009; Lamont et al., 2012). Using a different paradigm, Verhagen et al. have shown that GHSR knockout mice did not anticipate food when exposed to an activity-based anorexia model, in which mice are given free access to a running wheel and fed once per day for 2 h (Verhagen et al., 2011). Thus, most of these studies suggest that a disruption in the ghrelin signaling results in the lack of food anticipation behavior.

### Homeostatic eating in mice with both ghrelin and GHSR deficiency

In another study, significant differences in body weight (decreased), energy expenditure (increased) and locomotor activity (increased) were observed when both ghrelin and GHSR were deficient in mice (double knockout mice), whereas neither ghrelin deficiency nor GHSR deficiency independently affected energy balance under CHD feeding conditions (Pfluger et al., 2008). Thus, the authors speculated that additional components of the ghrelin signaling system might be present.

### Homeostatic eating in mice with tissue-specific GHSR expression

The GHSR-null mouse model with Cre recombinase-dependent reactivation of GHSR has been valuable in establishing the physiological roles of certain of ghrelin's brain targets (Zigman et al., 2005). In one study, we have used GHSR-null mice to assess the role of direct action of ghrelin on the ventral tegmental area (VTA) and other catecholaminergic neurons. We achieved this by crossing GHSR-null mice with mice containing Cre recombinase expression driven by the tyrosine hydroxylase (TH) promoter, resulting in mice expressing GHSR selectively in TH-containing cells normally programmed to express this receptor (Chuang et al., 2011). This selective GHSR expression did not affect body weight, but partially restored ghrelin-stimulated food intake to levels achievable in wild-type littermates (Chuang et al., 2011). In another study, we crossed GHSR-null mice with mice containing expression driven by the paired-like homeobox 2b (Phox2b) promoter, resulting in mice expressing GHSR selectively in specific hindbrain nuclei, including the nucleus of the solitary tract, dorsomotor nucleus of the vagus, area postrema, nucleus ambiguus and facial motor nucleus (Scott et al., 2012). In contrast to mice with catecholaminergic neuron-selective GHSR expression, mice with hindbrain-selective GHSR expression lacked ghrelin-induced acute food intake (Scott et al., 2012). Thus, as opposed to GHSR-expressing catecholaminergic neurons, GHSR-expressing hindbrain neurons are not sufficient to mediate the acute orexigenic effects of ghrelin.

### Homeostatic eating in mice with GHSR over expression in growth hormone releasing hormone neurons

Lall et al. generated a mouse model overexpressing GHSR in GH releasing hormone (GHRH) neurons using a rat GHRH cosmid promoter (GHRH-GHSR) (Lall et al., 2004). GHRH-GHSR mice had an increased post-weaning growth rate compared to wild-type littermates. When placed on HFD at 2 months of age, weight gain of GHRH-GHSR mice was similar to that of their wild-type counterparts, although fat pad mass trended lower. Food intake was not measured for individually-housed mice, however groups of GHRH-GHSR mice ate significantly more calories when fed HFD than when fed CHD, while groups of wild-type mice ate similar caloric amounts of HFD and CHD (Lall et al., 2004).

### Homeostatic eating in mice with goat deficiency

Two GOAT knockout mouse lines have been reported (Gutierrez et al., 2008; Zhao et al., 2010a). Using one of these lines, GOAT knockout mice demonstrated higher levels of desacyl-ghrelin compared to their wild-type littermates, and under CHD feeding had normal body weight and fat mass (Kirchner et al., 2009). However, when fed HFD, these GOAT knockout mice displayed significantly lower body weights compared to wild-type animals, despite not having significant changes in body composition. Challenging GOAT knockout mice with HFD rich in medium-chain triglycerides significantly reduced body weight and fat mass compared to similarly-treated wild-type littermates (Kirchner et al., 2009). Such was likely due to increased energy expenditure, since food intake in the GOAT knockout mice was higher (Kirchner et al., 2009). In contrast, the GOAT knockout mice generated by Zhao et al. had similar body weight, body composition and food intake as wild-type littermates, both on CHD and HFD (Zhao et al., 2010a). They also demonstrated similar weight loss curves when subjected to a severe caloric restriction protocol (Zhao et al., 2010a).

### Homeostatic eating in mice with deficiencies in ghrelin signaling and leptin

Ghrelin and leptin are both regulators of feeding, but have opposite effects on food intake. Unlike ghrelin, leptin increases with positive energy balance and inhibits food intake. Furthermore, leptin- and ghrelin-responsive central targets partially overlap (Zigman and Elmquist, 2003; Perello et al., 2012). To investigate the physiological roles of ghrelin and leptin signaling, and their potential interaction in regulating energy balance, Sun et al. crossed ghrelin knockout mice with leptin deficient (ob/ob) mice to generate mice lacking both ghrelin and leptin (ghrelin knockout ob/ob). Although they predicted that the ghrelin knockout ob/ob mice would have a leaner and relatively hypophagic phenotype, the double knockout mice instead displayed similar food intake and a modest increase in body weight as compared to ob/ob mice (Sun et al., 2006). Ma and colleagues generated mice with GHSR deletion on the ob/ob background (GHSR knockout ob/ob) (Ma et al., 2012). They found that food intake and body weights of GHSR knockout ob/ob mice were similar to those of ob/ob mice, possibly suggesting that the obesity in ob/ob mice is unrelated to unopposed ghrelin action.

## Ghrelin and hedonic eating

Hedonic eating is defined as food intake motivated primarily by pleasure. Hedonic eating also encompasses behaviors aimed at facilitating access to pleasurable food. Ghrelin is thought to increase the rewarding value of palatable foods. Recent studies show that ghrelin is able to regulate different aspects of hedonic eating. The two-food choice test is a conventional behavioral method for determining preference. In this test, rodents are offered a pair of different types of food simultaneously, and the consumed amount is measured for a defined time. Using this test, it has been shown that ghrelin administration shifts food preference toward diets rich in fat (Shimbara et al., 2004). Ghrelin administration also increases intake of palatable saccharin solution and preference for saccharin-flavored foods in mice (Disse et al., 2010). Similarly, rats treated with a GHSR antagonist consume less peanut butter and Ensure®, but do not change intake of CHD in a free choice protocol (Egecioglu et al., 2010). As will be discussed below, ghrelin administration also affects other models of hedonic eating, including the conditioned place preference (CPP) test and operant responding.

The central distribution of GHSRs within mesolimbic centers supports the likelihood of ghrelin playing a major role in hedonic eating. In particular, GHSR is highly expressed in dopaminergic VTA neurons, which project to the nucleus accumbens (NAc) and strongly drive reward behaviors (Perello and Zigman, 2012). Mesolimbic circuitries also may be indirectly regulated by cholinergic neurons located in the laterodorsal tegmental area (LDTg), which express GHSR and innervate the VTA (Dickson et al., 2010). In addition, we have recently shown that ghrelin action on food reward requires intact orexin signaling, although the neuronal circuits linking orexin neurons and ghrelin action remain unclear (Perello et al., 2010).

### Hedonic eating in mice with genetic deletion of GHSR

The use of mouse models deficient in GHSR have been instrumental in demonstrating an obligatory role for intact ghrelin signaling in various hedonic aspects of eating. Egecioglu et al. showed that GHSR knockout mice have a suppressed intake of the rewarding food in a free choice (CHD/rewarding food) paradigm and a reduced accumbal dopamine release induced by rewarding foods (Egecioglu et al., 2010). We recently subjected GHSR-null mice to a cue-potentiated feeding protocol, in which cue-potentiated feeding was demonstrated by enhanced intake of grain-based pellets selectively upon presentation of a positive conditioned stimulus (Walker et al., 2012). While wild-type mice demonstrated cue-potentiated feeding behavior, GHSR-null mice lacked this behavior. More specifically, GHSR-null mice displayed enhanced food intake in response to both positive and negative conditioned stimuli (Walker et al., 2012). This study supports pharmacologic studies suggesting a key role for intact ghrelin signaling pathways in establishing a specific positive cue-food association (Kanoski et al., 2013).

Altered eating behaviors are observed under stress as well as depression. In order to study the role of ghrelin in stress-associated hedonic eating, our group subjected both GHSR-null and wild-type littermates to a mouse behavioral model of chronic psychosocial stress and major depression, known as chronic social defeat stress (CSDS). Mice were challenged with ten daily bouts of social defeat by aggressive CD1 male mice, a protocol which results in an increase in plasma ghrelin (Lutter et al., 2008). Despite an increase in plasma ghrelin levels, GHSR-null mice failed to increase intake of CHD in response to chronic stress, unlike wild-type controls which increased their food intake. This suggests that activation of GHSR signaling is required for a stress-induced increase in caloric intake. In addition, stressed GHSR-null and wild-type mice were also tested in a food CPP test, which models a more complex, reward-related type of eating behavior. In the food CPP test, animals are conditioned to associate one chamber of the CPP apparatus with CHD and a second, visually and texturally distinct chamber with an equal calorie amount of a more pleasurable food, such as HFD. After conditioning, animals are given free access to both chambers in the absence of the food, and CPP is demonstrated when more time is spent in the chamber previously paired to the food reward. The CSDS-CPP protocol seems to model stress-based comfort food eating. Following exposure to the CSDS protocol, GHSR-null mice did not demonstrate CPP for HFD, whereas wild-type mice did (Chuang et al., 2011).

The above findings in GHSR-null mice confirm pharmacological studies; as administration of ghrelin and other means of achieving increases in plasma ghrelin (caloric restriction) both enable acquisition of food CPP (Perello et al., 2010; Disse et al., 2011), while administration of GHSR antagonists blocks food CPP (Egecioglu et al., 2010; Perello et al., 2010). Likewise, in our studies using mice subjected to a caloric restriction in which mice were limited to 80% of their usual daily food intake, GHSR-null mice did not demonstrate CPP for HFD, whereas wild-type mice did (Perello et al., 2010). Interestingly, when the study was repeated in mice exposed to a more intense calorie restriction protocol, in which mice were limited to *ad libitum* access to CHD for 4 h per day, no differences between genotypes were detected in the food CPP test. Thus, endogenous increases in GHSR signaling following stress as modeled with the CSDS protocol or under mild food restrictive conditions enable acquisition of CPP for HFD (Perello et al., 2010).

### Hedonic eating in mice with tissue-specific reactivation of GHSR

We also assessed food CPP performance following CSDS or as induced by administered ghrelin in mice with catecholaminergic selective GHSR expression, using the mice described above in section Homeostatic Eating in Mice with Tissue Specific GHSR Expression (Chuang et al., 2011). Such selective GHSR expression in TH-expressing cells was sufficient to mediate the ability of administered ghrelin or chronic stress to induce CPP for HFD.

### Hedonic eating in mice with goat deficiency

Recently, it was determined that GOAT-mediated acylation of ghrelin is a critical modulator of food reward. Following a 24-h food deprivation period, GOAT-deficient mice displayed an attenuated motivation for food in an operant responding model (Davis et al., 2012). This supports several pharmacologic studies in which ghrelin administration increased operant lever pressing for sucrose, peanut butter-flavored sucrose or HFD pellets in rodents, and in which GHSR antagonist reduced operant responding for sucrose solution (Perello et al., 2010; Finger et al., 2012; Landgren et al., 2012; Skibicka et al., 2012). GOAT knockout mice also showed a decreased hedonic feeding response as examined in a “dessert effect” protocol, wherein the intake of a palatable HFD “dessert” was assessed in calorically-sated mice (Davis et al., 2012). Furthermore, GOAT knockout mice displayed atypical transcriptional changes in orexin 1 receptor gene expression in the NAc, potentially implying the involvement of orexin signaling in these effects of ghrelin (Davis et al., 2012). Taken together, these results suggest that the motivation to obtain food is regulated by endogenous GOAT activity.

## Discussion

In this review, we have summarized the findings of ghrelin action on eating behavior as determined or confirmed using different mouse models with genetic manipulations of the ghrelin signaling system. Of note, the various transgenic mouse models more consistently demonstrated alterations in hedonic eating, while changes to homeostatic eating and body weight changes were more variable and subtle.

An important consideration when evaluating the eating-related phenotypes of the various transgenic models relates to differences between manipulations of ghrelin vs. GHSR vs. GOAT. For example, variable degrees of altered homeostatic eating, hedonic eating and body weight have been observed in ghrelin-deficient, GHSR-deficient, and GOAT-deficient models. Although the exact reasons for these differences are unclear, they could result from differences inherent to each of those molecules. For instance, GHSR possesses strong ligand-independent constitutive activity when studied *in vitro* (Holst et al., 2004; Holst and Schwartz, 2004), and thus phenotypic changes in GHSR-null mice could result from the combined loss of ghrelin binding and of baseline GHSR “tone.” Additionally, it has been proposed that ghrelin has other receptors besides GHSR, which could explain why simultaneous deletion of both ghrelin and GHSR result in a more severe phenotype than deletion of either alone (Holst et al., 2004; Pfluger et al., 2008). Furthermore, GOAT may have other substrates besides ghrelin, and thus its loss could directly affect more than just ghrelin bioactivity.

Another concern when evaluating these mouse models, as with any transgenic mouse models, is the possibility of functional redundancy or developmental compensation by another related system, which may mask the true effect of manipulation of the ghrelin system. While this has not yet been demonstrated specifically for the ghrelin system, it has been demonstrated for AgRP neurons that are thought to be crucial for ghrelin's effects on eating. Importantly, unlike the lack of significant body weight or food intake phenotypes in mice with genetic AgRP ablation achieved since inception or in neonates, rapid starvation ensues when AgRP expression is deleted in adult mice (Qian et al., 2002; Gropp et al., 2005; Luquet et al., 2005). Such a time-dependent effect of genetic AgRP deletion may be influenced by a time-sensitive capacity for the development of compensatory neurocircuits (Bouret et al., 2004; Luquet et al., 2005). Indeed, leptin's neurotrophic effects on hypothalamic development are restricted to a critical neonatal period (Bouret et al., 2004). Similar time-sensitive neurotrophic actions have been predicted for ghrelin (Steculorum and Bouret, 2011).

Further considerations when evaluating the effects of genetic engineering—in particular, those changes induced in standard transgenic models in which the transgene is randomly inserted into the genome—include the potential for the site of integration to affect tissue specificity, levels of transgene expression as well as expression of other genes present in the nearby genomic vicinity. For all these reasons, the use of the multiple mouse models generated using different genetic strategies and comparison of these mice to mice treated pharmacologically with agents targeting the ghrelin system is important for best determining the importance of the ghrelin system in eating behavior

It should also be noted that these various genetically-engineered mouse models targeting the ghrelin system additionally have been used to assess the importance of ghrelin action in domains other than those related to eating and body weight. For instance, GHSR-null mice have been used to demonstrate a key role for ghrelin action in minimizing depressive-like behavior following chronic stress (Lutter et al., 2008). Also, ghrelin-overexpressing, ghrelin-knockout, GHSR-knockout, GHSR-null and GOAT-knockout mice all demonstrate alterations in glucose homeostasis, with the most extreme phenotype—namely marked hypoglycemia and near death—appearing upon severe caloric restriction of mice with genetic deletion of either GOAT or ghrelin (Zhao et al., 2010a; Li et al., 2012).

These studies predict eating- and body weight-related phenotypes for people if they were to carry a genetic mutation engendering either ghrelin and/or GOAT overexpression or ghrelin, GHSR or GOAT deletion. Indeed, the extreme hyperphagia, early-onset obesity and debilitating food seeking behaviors associated with Prader-Willi Syndrome, in which ghrelin levels are found to elevated and abnormally regulated, represent much more extreme phenotypes than the mouse models of ghrelin over expression would predict (Feigerlova et al., 2008; Hinton et al., 2010). As of yet, no definitive examples of ghrelin, GHSR or GOAT deletion have been identified in people. However, other types of mutations—in the ghrelin gene and the GHSR gene—have been identified. One, the Leu72Met ghrelin polymorphism has been associated with binge eating disorder in small cohorts (Monteleone et al., 2007). Several GHSR polymorphisms have been found in individuals with short stature, which makes sense given ghrelin's ability to potently stimulate GH secretion. Using *in vitro* methods, these mutations result in loss of endogenous constitutive activity of GHSR, altered binding of ghrelin, decreased ghrelin-stimulated signal transduction, and/or decrease cell surface expression of GHSR (Wang et al., 2004; Pantel et al., 2006; Inoue et al., 2011). Interestingly, carriers of some of these GHSR mutations demonstrate an incomplete penetrance of overweight and obesity, although the relationship of the mutation and the body weight phenotypes have not been confirmed (Wang et al., 2004).

Given the crucial insight regarding ghrelin action on feeding and other domains provided by these mouse models, we would encourage further investigations using not only the currently available mouse models, but also novel genetically modified mouse models. For instance, a GHSR-conditional knockout mouse in which GHSR is eliminated exclusively from a single cell type or a single brain region by using the Cre-loxP technology is an exciting alternative. Additionally, the Cre-loxP system could be used to investigate the role of GHSR at specific stages of development by breeding GHSR floxed mouse lines with transgenic mice bearing a tamoxifen-dependent Cre recombinase expressed under the control of specific promoters, thus allowing the manipulation of GHSR to be controlled in a spatiotemporal-specific manner. In addition, mouse models containing some of the above-described ghrelin and GHSR polymorphisms described in individuals with binge eating disorder and families with short stature ± overweight/obesity would help clarify and causative role for the mutations in those conditions.

### Conflict of interest statement

The authors declare that the research was conducted in the absence of any commercial or financial relationships that could be construed as a potential conflict of interest.
